# Three-Stage Transitional Theory: Egalitarian Gender Attitudes and Housework Share in 24 Countries

**DOI:** 10.3389/fsoc.2021.700301

**Published:** 2021-11-05

**Authors:** Man-Yee Kan, Kamila Kolpashnikova

**Affiliations:** Department of Sociology, University of Oxford, Oxford, United Kingdom

**Keywords:** gender attitudes, housework, domestic division of labour, second demographic transition, gender, domestic work

## Abstract

How does the association between gender attitudes and housework share vary across countries and time? We examine the second demographic transition as it unmasks in the association between gender attitudes and housework participation. Using data of the 2002 and 2012 International Social Survey Programme (ISSP) for 24 countries, we find that the association between gender attitudes and housework share became stronger over time in most countries, signifying that the Second Demographic Transition was in place. The results also show that the association varied across the 24 countries, reaching an equilibrium in many but at different stages. Our findings suggest that equilibria in the domestic division of labour take various forms and paces in the ISSP countries.

## Introduction

Gender revolution in the labour market incites changes at home and vice versa. Although nowadays women still undertake more housework than men (e.g., Gershuny, 2000; Heisig, 2011; Hook, 2006; Hook, 2010; Kan, 2008a; Kan and He, 2018; Kan and Laurie, 2018; Kan et al., 2011; Kolpashnikova, 2018; Kolpashnikova and Kan, 2020), they are less confined to the traditional roles of the gendered housework division than before as revealed in the gradual reduction in their domestic work time over the years (Gershuny, 2000; Heisig, 2011; Hertog et al., 2021; Hook, 2006; Hook, 2010; Sullivan et al., 2018). Recent research generated more evidence that the housework share of women and men in heterosexual couples was converging in the industrial countries, albeit only slowly and intermittently (Altintas and Sullivan, 2017; Kan et al., 2011; Sullivan et al., 2018).

Scholars proposed that these changes in gender relations were a part of the Second Demographic Transition (SDT) (Lesthaeghe, 2010; van de Kaa, 2002), which also brought about the lowest fertility rates in history, increasing proportions of the elderly, and higher numbers of divorces. Esping-Andersen and Billari (2015) argued that the initial shockwave of the gender revolution and the departure from the traditional man-breadwinner woman-homemaker family specialization model brought about the decline in fertility, an increase in divorce rates, and above all, a realignment in gender relations at home. With time, new more egalitarian gender arrangements take root in the everyday lives of families because societies start to settle into the new equilibrium of non-traditional family forms when gender-egalitarian family arrangements are adopted by a critical mass of people and egalitarianism becomes normalized (Sullivan et al., 2018).

The demographic changes, described in Lesthaeghe (2010) and Esping-Andersen and Billari (2015), must be traceable in all individual activities affected by the shifts in gender ideology, including housework. Theoretical explanations of housework division focus on the resource-based frameworks and pay considerably less attention to the links with demographic theories. This is a major oversight considering that Esping-Andersen and Billari (2015) and Lesthaeghe (2010) postulate that it is the changes in gender ideology that drive demographic advances as well as the shifts in the division of domestic labour. Although housework participation and its association with gender attitudes have been examined in housework studies (Baxter, 1992; Bianchi et al., 2000; Coltrane and Ishii-Kuntz, 1992; Cunningham, 2005; Fuwa, 2004; Gazso-Windle and McMullin, 2003; Greenstein, 1996; Hu and Kamo, 2007; Kan, 2008a; Kan and Laurie, 2018; Kan et al., 2021; Kolpashnikova and Kan, 2020; Kolpashnikova et al., 2020; Lewin-Epstein et al., 2006), none of the studies explicitly connected their results with the SDT (Lesthaeghe, 2010). Our paper contributes by bridging housework theories with the multiple equilibria theories (Esping-Andersen and Billari, 2015) and develops a three-stage transitional theory to explain how the associations between gender attitudes and the gendered division of labour may take different forms in pre-transitional, transitional, and post-transitional societies.

This paper extends the theoretical expectations of the SDT by assessing the association between individual gender attitudes and housework participation across different welfare regimes. Our study is an illustration of how the changes in the association between gender attitudes and women’s and men’s housework share can be attributed to the theories of the SDT and multiple equilibria. The analysis uses the 2002 and 2012 International Social Survey Programme (ISSP) for 24 countries. Our key research questions are: 1) whether the differences between gender-egalitarian and gender-traditional women and men in their undertaking of housework share are exacerbated in the SDT; 2) whether there are welfare regime and regional differences in the association between gender egalitarianism and housework share, especially among the Scandinavian countries, which were previously identified as reaching post-SDT period. Our results indicate that the association between egalitarianism and housework share has strengthened between 2002 and 2012 for most regimes, suggesting that most countries were undergoing the SDT in this period. One notable exception is the Scandinavian countries, where the more traditional women and men were catching up with the egalitarian counterparts in their practice of housework division within households.

## Theoretical Framework

### Second Demographic Transition and Multiple Equilibria Theories

Lesthaeghe (2010) argued that the rapid decrease in fertility and the increase in union dissolution marked the SDT. Due to the prolongation of average education years and the value reorientation to individual self-actualisation and career-building rather than family, more women started postponing marriage and childbearing, which led to a precipitous fall in total fertility rates (TFR).

Conversely, Becker (1981) explained these demographic changes in terms of the convergence in women’s marketable skills with those of men. According to him, because women’s human capital increased while the returns to marriage and childbearing decreased, women were now more likely to postpone marriage or childbirth and to retain paid employment instead. Also, Oppenheimer (1988) postulated that the observed increase in the age at first marriage of women and men in Western developed countries is due to the shift in criteria of partner search. In the past, people looked for potential partners based on characteristics which were known at an earlier age, such as religion, ethnicity or outward appearance. However, education and earnings potential have become much more critical as criteria for a marriage partner. Given the improvement in women’s educational attainment and earnings and increases in economic uncertainties in the labour market, both women and men take more time to search for their partners. These have resulted in the postponement of first marriage and lower fertility rates (Zhou et al., 2017; Hertog, 2019; Kolpashnikova and Kan, 2021).

The recent research, however, presented evidence that the trends in TFR and divorce were reversing: in a few more gender-egalitarian societies, particularly in the Scandinavian region, the TFR began to increase (Lesthaeghe, 2010). Thus, the association of economic development and TFR reversed for those countries and became positive. The resulting rift between theory and empirical findings inspired new developments within the two competing frameworks.

First, Lesthaeghe (2010) extended his theory of the SDT by adding explanatory factors contributing to the reversal of the overall trend in selected countries. He argued that such reversal in TFR is only attainable in societies with higher levels of gender egalitarianism in most spheres of life including the labour market and the home, as well as an advanced system of social benefits and of policies helping women and men to balance work and family such as universal access to childcare facilities and generous paid paternity and maternity leaves.

On the other hand, Esping-Andersen and Billari (2015) challenged Lesthaeghe’s ideas that the reversal can happen only given certain criteria, specifically the contention that the institutional welfare support and progressive family policy are fundamental. The evidence that they provided was that the reversal was also apparent in the rest of the world where institutional support was not historically developed, such as in Southern European countries and the United States. These countries did not experience a period of protracted sub-replacement TFR, as Lesthaeghe (2010) predicted. Instead, Esping-Andersen and Billari (2015) contended that the period of low fertility rates was only a temporary shock to the previous old equilibrium in the traditional family relations marked by specialization of men in the labour market and women at home. However, as new family forms emerge such as dual-earners and women-breadwinner households, societies will settle into a multiple-equilibria model where new family forms will establish new relations between family members distinct from the old traditional relations equilibrium. Over time and with generational change, the whole system will stabilize into this new set of equilibria.

Trends around the world support the latter theory. For example, Myrskylä et al. (2009) found that among countries with the highest human development index (HDI), such as Norway, the Netherlands, the United States, the United Kingdom, Denmark, Germany, among others, the TFR reversed and became positive in between 1975 and 2005. Moreover, studies showed that there was a reversal in fertility among people of higher socioeconomic status in many advanced economies, especially in Scandinavia (Hoem, 1997; Lyngstad, 2004; Kravdal and Rindfuss, 2008). All the above international studies suggest that there occurred a reversal in fertility rates and marriage stability among the higher socioeconomic stratum, which may eventually spill over to the rest of the population.

Furthermore, following the multiple-equilibrium perpective, gender roles may settle into varying levels of egalitarianism from the transitional period of SDT, depending on social norms and welfare policies. For example, Esping-Andersen et al. (2013) analysed time diary data of Denmark, the United Kingdom and Spain and found that gender roles are egalitarian in both paid work and domestic work in Denmark, are gender traditional in Spain, and are at a stage of “unstable equilibrium” in the United Kingdom, where there are few traditional male-breadwinner families but the division of paid work and domestic work is gender unequal.

### Gender Ideology and the Division of Housework

The SDT also reflects in daily activities. Driven by the increase in egalitarian gender relations both in the labour market and at home, women and men re-adjust their day-to-day lives to accommodate the new gender ideology. It becomes increasingly normative for a woman to be employed, as well as for men, to do housework. For example, the British Social Attitudes Surveys reveal that in the United Kingdom, the percentage of population supporting the view that “a man’s job is to earn money; a woman’s job is to look after the home and the family” fell from 48% in 1987, to 13% in 2012 and to only 9% in 2017. 53% of the population agreed that “both the man and the woman should contribute to household income” in 1989. The figure rose to 62% in 2012, and to 72% in 2017 (Huchet-Bodet et al., 2019).

Egalitarian gender ideology concomitant with the SDT contributes to a more equitable division of housework. As Davis and Greenstein (2009) summarized it, studies consistently showed that more egalitarian women were doing less housework, whereas men with more egalitarian views were more likely to take on housework than traditional men (Baxter, 1992; Coltrane and Ishii-Kuntz, 1992; Greenstein, 1996; Bianchi et al., 2000; Gazso-Windle and McMullin, 2003; Fuwa, 2004; Nordenmark, 2004; Cunningham, 2005; Lewin-Epstein et al., 2006; Hu and Kamo, 2007; Kan and Laurie, 2018). Housework research, however, rarely connected the association between gender ideology and housework sharing with the SDT and the multiple equilibria theory.

On a closer look, most studies analysed in Davis and Greenstein (2009) and discussing the association between gender attitudes and housework participation reported somewhat mixed results. Hu and Kamo (2007) found a significant positive association between Taiwanese men’s gender egalitarianism and participation in housework but the association for Taiwanese women was not significant. Cunningham (2005) reported similar results for American men and women. Using 1994 ISSP data, Fuwa (2004) showed that men’s more egalitarian gender attitudes (EGA) contributed to a more equitable division of housework but did not test the association for women. Coltrane and Ishii-Kuntz (1992) found that gender traditionalism was significantly associated with less housework only for a specific group of American men—those who delay parenthood.

Similarly, Greenstein (1996) found that in the United States, egalitarianism was associated positively with housework participation only for husbands with already more egalitarian gender ideology. Conversely, Lewin-Epstein et al. (2006) reported that German and Israeli egalitarian women did significantly less housework than traditional ones, yet for men, the results were not significant and not in the expected direction. Gazso-Windle and McMullin (2003) established similar results for Canadian women and men, Baxter (1992)—for Australian women and men, Bianchi et al. (2000)—for American women and men. Only in one study, Kan (2008a), the results reported unequivocally that traditionalism was significantly associated with women’s increase and men’s decrease in housework time in the United Kingdom.

The mixed findings can be accounted for by the lagged adaptation to new gender relations (Gershuny et al., 1994). With the gradual erosion of gendered expectations, especially within the 20^th^ century, the traditional man-breadwinner woman-homemaker specialization model changed. Increasingly more families pushed to be dual-earners; more men shared housework responsibilities than before. The process of egalitarian gender socialization spread wider in families, in schools, at the workplace, and became normalized for all human activity (Davis and Greenstein, 2009). However, the transition to new family arrangements other than the traditional man-breadwinner woman-homemaker model needed time to adjust with the SDT and new equilibria in gender ideology. This ‘lagged adaptation’, envisioned by Gershuny et al. (1994) for men, can be responsible for the mixed and inconclusive results of the previous studies.

The ‘lagged adaptation’ or lagged alignment between gender ideology and housework participation can also be conflated with period and cohort effects because socialization is also dependent on period and cohort effects (Davis and Greenstein, 2009). Younger generations are socialized in a more egalitarian way than older generations; thus, they are expected to share housework in a more egalitarian way (Brewster and Padavic, 2000). Because of the period effect (Brewster and Padavic, 2000; Ciabattari, 2001; Carter and Borch, 2005), the gender ideologies might be just a reflection of a new era, rather than the true association between housework participation and gender ideologies, to name a few alternative explanations, which need to be weeded out.

### Three-Stage Transitional Theory

**FIGURE 1:**
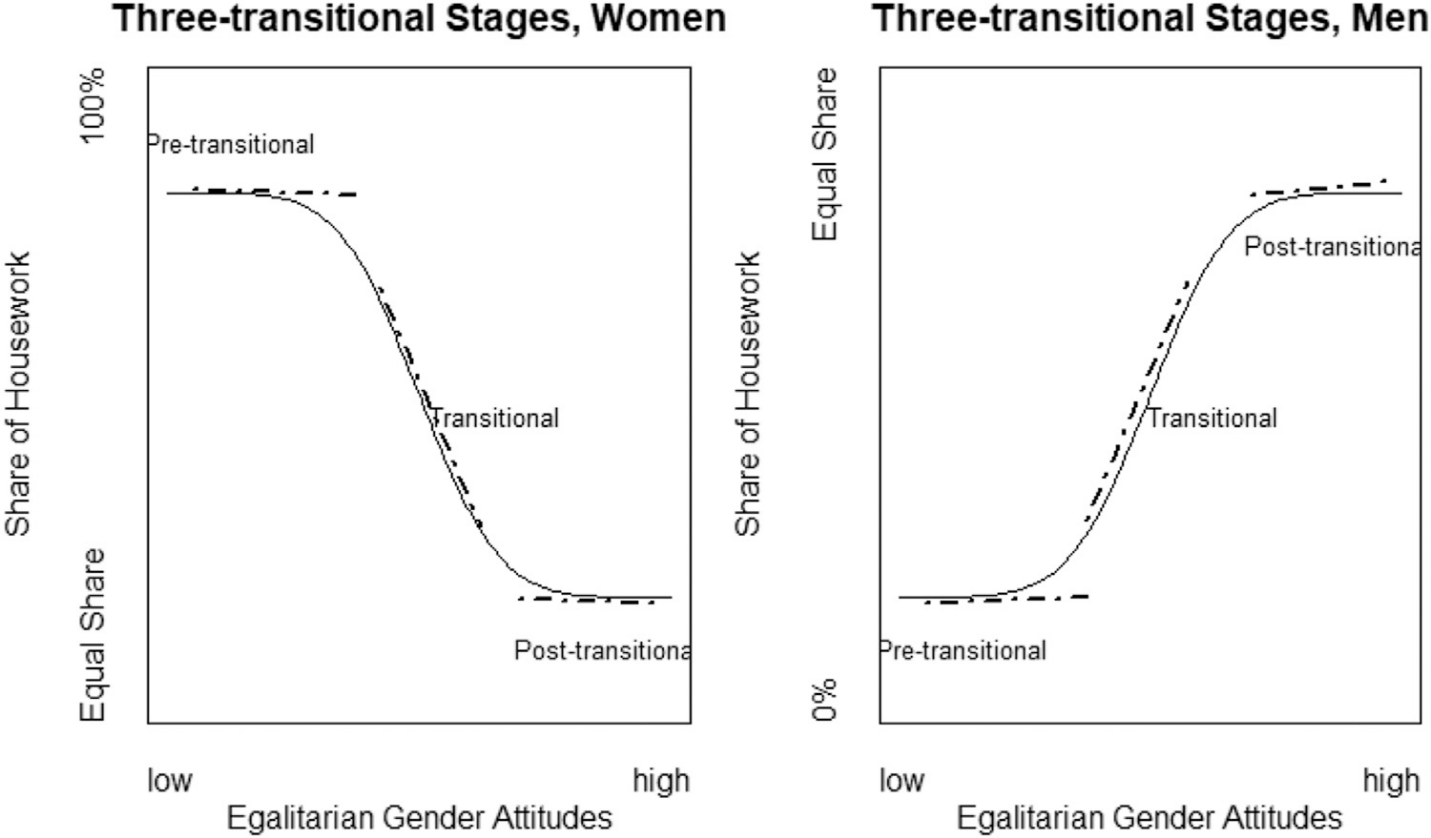
Three-stage transitional theory of the association between gender attitudes and housework share.

We postulate that the processes that societies undergo under the SDT can be separated into three stages: pre-transitional, transitional, and post-transitional. Applied to housework activities, the transition process can be tracked in the association between gender attitudes and housework share. Figure 1 shows the theoretical expectations about the change in the association between gender attitudes and housework share for women (left panel) and men (right panel).

Pre-transitional state identifies a prolonged historical period of the traditional division of labour, where family work division equilibrium was maintained by specialization: women did most of the housework (higher average level of housework share) and men took on paid work activities (lower average level of housework share) (Esping-Andersen and Billari, 2015). In this stage, the differences between housework share assumed by more egalitarian women compared to more traditional women are minimal. Thus, the slope of the association between egalitarianism and housework share for the pre-transitional stage remains relatively flat for both women and men.

When societies enter the SDT stage, women and men adopt more egalitarian attitudes (Lesthaeghe, 2010). However, the level of adoption varies according to the level of gender egalitarianism in attitudes and housework participation. The behaviour of more egalitarian women and men aligns better with their attitudes, whereas traditional women and men lag in the adoption of more egalitarian attitudes and behaviours. This creates starker differences between egalitarian and traditional women and men, due to the lagged adaptation phenomenon (Gershuny et al., 1994; Esping-Andersen and Billari, 2015). Thus, in the transitional stage, the slope of the association between egalitarianism and housework share is steeper, reflecting the sharper differences between egalitarian and traditional women and men.

Eventually, however, a transitional society enters the multiple equilibria stage with new family forms demonstrating more egalitarian relations between women and men (Esping-Andersen and Billari, 2015). In this stage, the previously lagging more traditional women and men catch up with their egalitarian counterparts in housework sharing, and the differences become less distinct compared to the previous stage. Thus, in the post-transitional stage, the slope of association between gender attitudes and housework share also flattens out for both women and men. This stage can happen at different levels of housework sharing in different countries. To recap, the multiple-equilibrium perpective suggests that gender roles may settle into varying levels of egalitarianism. We should note that here we focus on changes in the association between gender attitudes and housework participation, rather than changes in gender attitudes or housework participation only.

This process, however, can as well be non-linear due to political, societal, and economic shocks. For example, after the collapse of the Soviet Union, post-Soviet regime countries underwent a transition to more traditional gender relations as a result of the decline in institutional provisions and benefits such as universal access to nurseries and kindergartens (Pascall and Manning, 2000), the abolishment of state ideology, including the doctrine of the ‘Soviet women-workers’ capable to work on par with men (Atwood, 1999), the revival of religion promulgating more orthodox gender values, and because of globalization and the diffusion of more traditional gender attitudes common in other countries outside of the Soviet Union. Therefore, political, economic, and social shocks can cause a reversal at all stages of the process, depicted in Figure 1.

### Hypotheses

Generally speaking, egalitarian gender attitudes are associated with less housework among women and more housework among men. As it takes time to achieve symmetrical gender roles from traditional ones, we expect to find that all countries of the ISSP surveys but Scandinavian countries are at the transitional stage of the SDT between 2002 and 2012.

Hypothesis 1. The association between gender attitudes and housework became stronger between 2002 and 2012 for both men and women in all countries of the ISSP surveys except Scandinavian countries.

After the transitional period, societies are expected to settle in a new system of multiple family forms and archetypes of family and gender relations. In post-transitional society, we expect that more traditional women and men will catch up with egalitarian ones in their behaviour, including participation in housework. Therefore, the differences between traditional and egalitarian women and men in their housework activities would decrease. We expect that there are differences among the countries in their paces of going through the transitional stages.

On the one hand, the association between gender attitudes and housework share will have become weaker between 2002 and 2012 in countries where the SDT has already occurred (e.g., the social-democratic regime countries) because traditional women and men catch up with more egalitarian counterparts in these countries. On the other hand, the association remains weak in 2012 (and in 2002) in countries which are presumed to be in the pre-transitional stage. Thus, we expect to find weaker differences between egalitarian and traditional women and men in Scandinavian countries, where the previous literature has established that the new equilibrium of the post-transitional stage has been reached (Esping-Andersen and Billari, 2015). Conversely, we expect sharper differences between egalitarian and traditional women and men in countries, which have only started to undergo the transitional period such as Southern European countries. Additionally, we expect more stable systems and fewer differences between egalitarian and traditional women and men in countries outside of the Western world, where the processes might not yet have started or may have a different cultural trajectory, such as in countries in East Asia, Eastern Europe, and Latin America.

Hypothesis 2: The association between gender attitudes and housework became weaker in Scandinavian countries as they have already reached the post-transitional stage of the SDT.

Given differences in gender norms, societal and policy contexts, in line with the multiple equilibrium perspective, there are variations in the pace of change in the association between gender attitudes and housework among different countries.

Hypothesis 3: The change in the association between gender attitudes and housework between 2002 and 2012 varied among the countries.

## Data, Measures and Analytical Strategies

In our analyses of the association between egalitarian gender attitudes and housework share, we use cross-national data of the ISSP 2002 and 2012 Family and Changing Gender Roles (ISSP Research Group, 2009). Sample sizes vary across the countries, but the focus is on the individuals above 18 years old. The sample included only the states, which had all variables of interest present in both survey years. We had to drop a few counties in the process. For example, Finland and the Netherlands data did not contain personal income information at least 1 year, so they had to be removed from the sample. These decisions, of course, present some limitations in terms of representation of the regimes. For instance, the social-democratic regime countries are here based on three countries: Denmark, Norway, and Sweden. Additionally, although there were data for both years for Austria and Belgium, these two countries were not included in the sample because their surveys did not include questions about housework participation and its share in 2002. In one instance, Bulgaria, information on respondents’ children in 2002 was not available, and we decided to drop Bulgaria as well.

Following previous studies (e.g., Esping-Andersen, 1990; Sainsbury, 1999; Kan et al., 2011), we group countries into welfare regimes and regions based on the public welfare provision, gender ideologies, and the level of social equity. First, in Social Democratic regimes or the Scandinavian region (Denmark, Norway, and Sweden), gender equity in employment is high, and generous parental and paternal leave policies are in place to promote dual-earner families. In Liberal regimes (Australia, the United Kingdom, and the United States), the state provides minimal welfare support. Childcare services are primarily provided by the market. Women are expected to take a secondary role as breadwinner in the family and the major caring role in the family. In Southern European regimes (Spain and Portugal), social policies rely on traditional family networks as the source of care support and therefore the level of gender inequality in domestic work and employment is high. In Conservative regimes (France, Germany, and Switzerland), the state takes a subsidiary role in welfare provision. There are generous parental leave policies, but a male breadwinner role and a female carer role are assumed in social policies.

The welfare regimes in the rest of the ISSP countries covered in this study were developed at a later stage. In East Asian regimes (Japan and Taiwan), the welfare policies were built upon the Confucian ideology which emphasizes family ties and traditional gender roles (Sung and Pascall, 2014). Public social expenditure in these countries is the lowest among the Organisation for Economic Cooperation and Development (OECD) countries (Gauthier, 2016). The Eastern European regimes (the Czech Republic, Hungary, Poland, Slovakia, Slovenia, and former Soviet Union republics) are characterised by a relatively high level of female employment and an extensive public sector. They have mixed characteristics of the Conservative and the Social Democratic welfare regime types (Fenger, 2007). In Latin American regimes (Mexico and Chile), the state provides limited welfare support to the family. Public spending on children’s benefits is below the average of the OECD countries. Women’s labour force participation rate is low, and the domestic division of labour is highly gender unequal (Blofield et al., 2021).

From results of cluster analysis, the Philippines matched better with the Latin American countries than with other Asian countries. For the Philippines and Israel, we performed a cluster analysis to define country typologies, where these two countries would fit better. The model outputs can be found in Supplementary Appendix 1. The results helped us to classify Israel with the Liberal regimes and the Philippines with Mexico and Chile. Although there were differing results, especially for the Eastern European countries, we decided to keep them in a separate group because of the shared historical trajectory and to rely on the earlier theoretical frameworks, such as that of the welfare regimes (Esping-Andersen, 1990; Sainsbury, 1999), to cluster the rest.

### Dependent Variable

We use the share of self-reported weekly hours spent on housework in the combined weekly housework hours of respondents and their spouses to construct the dependent variable of housework share. The stylised surveys like the ISSP often suffer from the social desirability bias, and researchers often recommend using time-use measures of housework participation (Kan, 2008b; Kan and Pudney, 2008) such as the Multinational Time Use Survey and the Harmonised European Time Use Survey. However, time use surveys do not usually collect instruments measuring gender ideology as the ISSP does.

The ISSP collects surveys in a range of European countries and beyond, including some in Latin America. We opted to use the measure of housework share instead of absolute hours spent on housework because using this measure is more apt for inter-country comparisons.

### Key Independent Variables

We aggregated seven questions regarding gender attitudes, which were available in both 2002 and 2012 surveys to construct the egalitarian gender attitudes (EGA) scale: “To what extent do you agree or disagree with the following statement:” 1) “A working mother can establish just as warm and secure a relationship with her children as a mother who does not work;” 2) “A preschool child is likely to suffer if his or her mother works;” 3) “All in all, family life suffers when the woman has a full-time job;” 4) “A job is all right, but what most women really want is a home and children;” 5) “Being a housewife is just as fulfilling as working for pay;” 6) “Both the man and woman should contribute to the household income;” 7) “A man’s job is to earn money; a woman’s job is to look after the home and family”. The response options ranged from 1) ‘strongly agree’ to 5) ‘strongly disagree’. The Spanish questionnaire differed from those in other countries on this scale. Instead of the option 3 (3 = ‘neither agree nor disagree’) Spanish surveys had a response choice ‘can’t choose’, which we re-coded as 3) as well. Based on the preliminary item consistency analysis, we reverse coded items 1 and 6. The Cronbach’s alpha for the resulting scale was 0.73. The item correlations table can be found in Supplementary Appendix 2. Gender egalitarianism measured using this scale has increased in the total analytical sample from 2002 to 2012 (Tables 1, 2).

**TABLE 1:** Means of main variables women in ISSP countries.

	HW share, 2002	HW share, 2012	EGA, 2002	EGA, 2012	Paid work, 2002	Paid work, 2012	Dependency, 2002	Dependency, 2012
Australia	71.125	64.115	3.408	3.489	18.881	24.769	2.373	4.822
Chile	82.763	77.753	2.673	2.974	20.201	18.231	5.092	4.964
Czech	82.461	70.374	2.785	3.310	24.512	29.607	2.359	4.674
Denmark	67.587	62.606	4.043	4.151	36.763	34.706	4.643	4.472
France	78.341	72.007	3.706	3.809	29.844	28.126	4.718	4.587
Germany	70.965	71.694	3.961	3.913	28.813	22.095	4.637	4.634
Hungary	74.134	75.037	2.986	2.876	24.602	19.094	4.792	4.519
Israel	71.090	72.893	3.577	3.346	31.735	25.553	4.355	4.247
Japan	91.983	87.342	3.364	3.360	20.609	21.569	5.888	5.945
Latvia	66.196	67.514	3.128	2.938	34.342	30.772	4.315	4.007
Mexico	69.716	72.251	2.928	2.754	31.881	17.224	3.531	4.056
Norway	73.369	67.363	3.774	4.024	32.801	36.925	4.875	4.571
Philippines	65.625	68.261	2.857	2.811	41.545	16.160	4.255	5.620
Poland	65.243	65.713	3.198	3.226	42.292	25.458	3.991	4.622
Portugal	80.828	79.405	3.186	3.516	36.967	28.568	4.420	4.606
Russia	66.928	66.467	2.972	2.962	35.324	25.662	4.440	4.558
Slovakia	65.903	70.816	3.103	3.176	30.245	27.373	2.653	4.491
Slovenia	66.301	73.008	3.146	3.692	33.229	25.285	3.571	4.285
Spain	73.652	71.728	3.597	3.635	30.577	23.005	4.630	4.885
Sweden	64.971	61.719	3.911	4.217	35.197	32.920	4.493	4.512
Switzerland	72.611	73.459	3.389	3.370	29.740	21.351	3.089	3.684
Taiwan	73.328	73.081	3.223	3.135	43.325	28.940	4.390	5.120
United Kingdom	72.499	70.638	3.453	3.567	26.008	21.533	4.770	4.815
US	69.843	66.440	3.399	3.312	23.931	24.292	4.476	4.418
Total	72.823	70.420	3.406	3.450	31.122	25.518	4.509	4.678
*N*	4,321	5,408	4,321	5,408	4,321	5,408	4,321	5,408

HW—‘Housework’, EGA—‘Egalitarian Gender Attitudes’. Full tables with standard deviations are available upon request.

**TABLE 2:** Means of main variables men in ISSP countries.

	HW share, 2002	HW share, 2012	EGA, 2002	EGA, 2012	Paid work, 2002	Paid work, 2012	Dependency, 2002	Dependency, 2012
Australia	32.373	37.374	2.938	3.313	39.173	42.496	2.327	2.928
Chile	22.913	23.172	2.705	2.806	52.052	45.707	2.343	2.349
Czech	29.366	33.056	3.170	3.262	38.605	40.214	3.049	3.209
Denmark	37.648	41.865	3.908	4.109	42.535	39.455	3.061	3.277
France	31.442	36.342	3.389	3.704	40.833	38.484	3.058	3.062
Germany	27.626	31.716	3.568	3.686	43.968	40.756	2.648	2.773
Hungary	27.799	30.810	2.974	2.915	41.482	26.524	3.141	3.499
Israel	28.719	30.432	3.339	3.223	42.779	40.872	2.897	3.173
Japan	9.936	15.465	3.205	3.392	54.230	48.188	2.016	2.036
Latvia	38.194	36.604	2.984	2.928	47.046	39.157	3.054	2.948
Mexico	32.160	33.548	2.845	2.812	51.316	42.549	2.516	2.852
Norway	33.784	40.775	3.630	3.792	42.358	43.282	2.891	3.100
Philippines	41.014	35.444	2.882	2.793	45.355	35.916	2.307	3.121
Poland	37.310	35.423	3.028	3.118	49.450	38.805	2.574	2.847
Portugal	24.039	29.065	2.907	3.365	39.654	40.155	3.014	3.264
Russia	36.382	35.790	3.000	2.925	41.250	39.795	3.248	2.766
Slovakia	28.040	34.783	2.780	3.049	44.529	34.715	2.412	3.139
Slovenia	13.553	27.425	2.843	3.608	44.567	36.500	2.667	3.353
Spain	27.325	31.839	3.378	3.496	40.799	35.961	2.477	3.001
Sweden	38.094	41.174	3.734	3.886	40.946	40.592	3.211	3.140
Switzerland	25.952	29.491	3.072	3.279	45.584	43.510	2.527	2.634
Taiwan	24.298	27.520	3.009	3.074	48.792	46.055	2.532	2.836
United Kingdom	34.794	39.530	3.266	3.493	43.626	38.283	2.787	3.180
US	39.988	35.928	3.275	3.213	42.987	40.372	2.815	3.084
Total	31.087	33.532	3.210	3.340	44.729	40.289	2.731	2.973
*N*	3,949	4,634	3,949	4,634	3,949	4,634	3,949	4,634

HW—‘Housework’, EGA—‘Egalitarian Gender Attitudes’. Full tables with standard deviations are available upon request.

**FIGURE 2:**
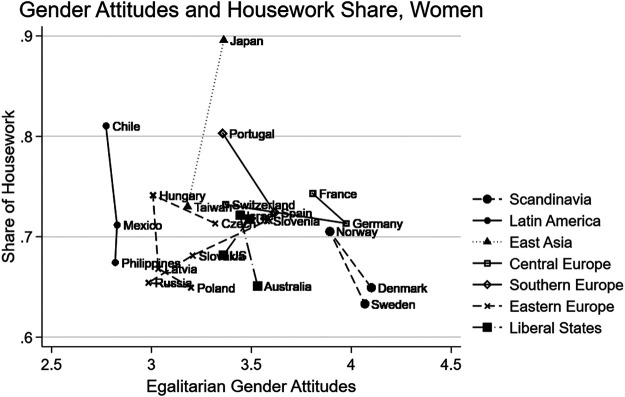
Egalitarian gender attitudes and housework share country means, women.

**FIGURE 3:**
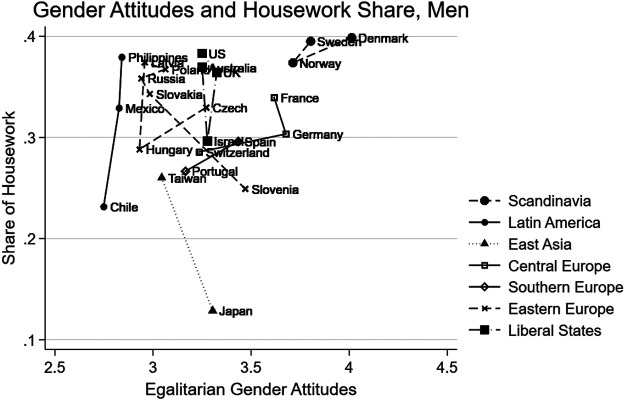
Egalitarian gender attitudes and housework share country means, men.

Figure 2 plots the average housework share on the *y*-axis and the mean EGA on the *x*-axis among women by country, and Figure 3—among men based on a pooled sample of the 2002 and 2012 ISSP data. We also connected the countries in the same region/regime by lines. Overall, Figure 3 shows that counties in the same regime or region tend to cluster together. As reported in the previous literature, women in Scandinavian countries report the highest level of egalitarianism and do a lower share of housework compared to women in other regions. In Liberal regime and Eastern European countries, women take on a smaller share of housework but compared to Eastern Europe, women in Liberal states also share higher levels of EGA.

Conversely, in Central European countries, women have as egalitarian attitudes as women in Liberal regime countries. However, the housework share that they undertake at home is higher than that among women in Liberal-regime countries. Figure 2 also shows that Southern European, East Asian, and Latin American women shoulder a higher share of housework responsibilities than women in other regimes, particularly in Japan. The housework share of Japanese women is the highest among all countries (Figure 2).

Among men, the patterns in Figure 3 are a mirror image of that of women’s in Figure 2. Figure 3 shows that Scandinavians report, on average, the highest levels of EGA and follow their values in their behaviour by taking on the highest share of housework compared to other men. The least egalitarian attitudes are reported among Latin American men. Japanese men are a single outlier; they do the least share of housework compared to other men while sharing comparatively higher gender-egalitarian attitudes for their level of housework division.

### Control Variables

The multivariate models control for employment status of the respondent and the spouse, level of dependency, whether outsourcing of housework is used, years in education, whether the respondent is married or cohabiting, number of children under 17, household size, age cohort, and country.

Considering diverging levels of personal and household incomes among ISSP countries, we decided to harmonize income variables. We recoded income variables into categories by quartiles, in each nation by year. Thus, the first quartile (which is also used as the reference category in the models) represents the group with the lowest 25% of income in a country in either 2002 or 2012, whereas the fourth quartile includes the upper 25%.

Respondent reported own and spousal weekly average work hours and employment status. The ISSP measured paid work time in hours spent in a regular week, capped at 96 h. The employment status variable is represented by a dummy variable (1 = ‘employed’; 0 = ‘otherwise’). Tables 1, 2 show that women and men’s weekly work hours decreased between 2002 and 2012. We included the measure of education in years. The level of economic dependency uses the ISSP item reporting the partner in a couple with higher income. This variable ranges from 1 (‘spouse has no income’) to 7 (‘I have no income’).

We also control for housework outsourcing to people that are not the married couple within and outside the household (1 = ‘housework outsourced’; 0 = ‘otherwise’). We divided age into seven cohorts; the cohort of those born in between 1978 and 1982 is the reference category.

We also included household composition variables into the models. These variables are the number of children and the number of household members. All models include a control variable of country and survey year because many countries might begin the transitional stage as well as the post-transitional stage at different levels of housework division between spouses. Thus, the intercepts may vary not only for regions but also among countries within the regions. Therefore, we decided to include the country dummy variables into the models as well.

### Sample Selection

The total ISSP sample before the sample selection contains 108,392 observations. First, we excluded countries where the information for any of the independent variables was not available. This truncation left us with 68,549 observations in 24 countries. The main country-level sample included 2 years: 2002 and 2012. The sample further was restricted to people between 20 and 59 years of age, which included 43,231 observations. We capped the number of children at 6, household size at ten people, and education at 20 years. After removing observations with missing values, the total sample contained 19,609 observations.

### Models

We utilize two-level random-intercept random-slope models separately for women and men, and the second level is regimes/regions. Our data comprised of individuals clustered by welfare regimes/regions, where each regime/region has an individual intercept. We then analyse the inter-regional variation in the association between gender-egalitarian attitudes and housework share among women and men, using random slopes for estimating the association. Additionally, we conducted a few robustness checks using OLS regression models with controls for regions and countries.

We use the multi-level random intercept—random slopes estimation in all our models. Due to heterogeneity among countries, all models control for country and survey year.

We have checked the robustness of the results using other techniques such as OLS regression separately for each region with country dummies and country interactions with EGA variable. The results of the robustness checks are available upon request.

## Results

### Overall Results

**FIGURE 4:**
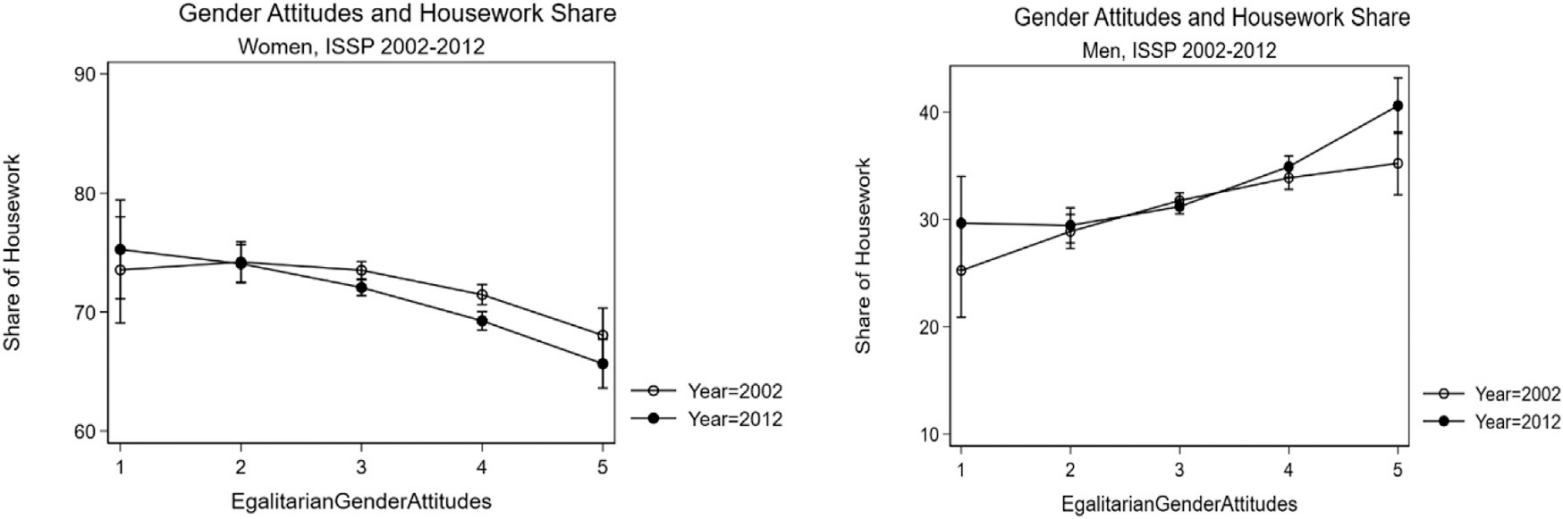
Marginal Means of Egalitarian Gender Attitudes on Housework Share based on Models (5) in Tables 3, 4, 95% Confidence Intervals.

Tables 3, 4 summarise the outputs for random intercept-random slope regression estimates for the year 2002 (Model 1), year 2012 (Model 2), pooled 2002–2012 without control variables (Model 3), pooled model with year interactions (Model 4), and pooled model with curvilinear association tested for the EGA variables and its interaction with the year variable (Model 5). Model 5 is later used to produce marginal means plots in Figure 4.

**TABLE 3:** Random intercept—random slopes estimates for housework share among women, ISSP 2002–2012.

	Model (1) 2002	Model (2) 2012	Model (3) combined	Model (4)	Model (5)
EGA	−1.784^***^ (0.427)	−2.700^***^ (0.497)	−2.306^***^ (0.424)	−1.865^***^ (0.483)	3.712 (2.357)
Year: 2012			−2.059^***^ (0.352)	0.909 (1.533)	6.094 (5.339)
2012 # EGA				−0.872^*^ (0.438)	−4.196 (3.212)
EGA # EGA					−0.839^*^ (0.348)
2012 # EGA # EGA					0.503 (0.469)
Controls	Yes	Yes	Yes	Yes	Yes
Constant	75.826^***^ (3.042)	70.918^***^ (2.796)	73.452^***^ (2.136)	72.050^***^ (2.258)	63.047^***^ (4.272)
N	5,396	5,408	10,804	10,804	10,804
Log-likelihood	−23003.686	−23173.736	−46214.546	−46212.657	−46209.181
Chi Square	1215.594	865.360	1909.243	1902.340	1999.194
D.f.	44	44	45	46	48

Standard errors in parentheses.^+^ *p* < 0.10, ^*^ *p* < 0.05, ^**^ *p* < 0.01, ^***^ *p* < 0.001. Models also control for employment status of the respondent and the spouse, level of dependency, outsourcing, household and personal income quartiles, years in education, being married, having children under 17, household size, age cohort, and country. The full outputs are available in Supplementary Appendix 3.

**TABLE 4:** Random intercept—random slopes estimates for housework share among men, ISSP 2002–2012.

	Model (1) 2002	Model (2) 2012	Model (3) combined	Model (4)	Model (5)
EGA	2.677^***^ (0.451)	2.897^***^ (0.776)	2.907^***^ (0.457)	2.223^***^ (0.530)	4.959^+^ (2.696)
Year: 2012			0.172 (0.397)	−4.041^*^ (1.725)	10.641^+^ (5.859)
2012 # EGA				1.282^*^ (0.511)	−7.995^*^ (3.596)
EGA # EGA					−0.416 (0.408)
2012 # EGA # EGA					1.392^**^ (0.537)
Controls	Yes	Yes	Yes	Yes	Yes
Constant	31.279^***^ (3.702)	19.102^***^ (3.251)	24.295^***^ (2.378)	26.436^***^ (2.523)	22.107^***^ (4.787)
N	4,171	4,634	8,805	8,805	8,805
Log-likelihood	−17848.415	−19834.218	−37730.291	−37727.149	−37723.173
Chi Square	1262.698	929.516	2090.582	2099.129	2124.940
D.f.	44	44	45	46	48

Standard errors in parentheses. ^+^ *p* < 0.10, ^*^ *p* < 0.05, ^**^ *p* < 0.01, ^***^ *p* < 0.001. Models also control for employment status of the respondent and the spouse, level of dependency, outsourcing, household and personal income quartiles, years in education, being married, having children under 17, household size, age cohort, and country. The full outputs are available in Supplementary Appendix 3.

Women and men with more EGA are more likely to share housework equally between spouses. Results in Tables 3, 4 confirm that EGA is associated significantly with housework share for both women and men. The results for women and men are consistent with Hypothesis 1. The association between gender attitudes and housework share remains on a statistically significant level for women and men when we introduce an interaction term with the period effect (Model 5 in Tables 3, 4). The interaction terms are on a significant level as well. The findings, therefore, indicate that the association between egalitarianism and housework participation has changed in between 2002 and 2012. These results confirm the previous findings on the association between gender attitudes and housework participation (Baxter, 1992; Coltrane and Ishii-Kuntz, 1992; Greenstein, 1996; Bianchi et al., 2000; Gazso-Windle and McMullin, 2003; Fuwa, 2004; Cunningham, 2005; Lewin-Epstein et al., 2006; Hu and Kamo, 2007; Kan, 2008a; Kan and Laurie, 2018; Kolpashnikova and Kan, 2020; Kan et al., 2021).

The effects of gender attitudes on housework share are a little stronger for women in 2012 compared to 2002. In 2012, an average woman with the highest score on the egalitarianism scale did 11% less of the housework share than an average woman with traditional gender attitudes (Model 2 in Table 3, b = −2.700, SE = 0.497), an average man with more EGA did about 10% more than an average man with traditional gender attitudes (Model 2 in Table 4, b = 2.897, SE = 0.776). Overall, however, the results are not that straight forward. Thus, we find only marginal evidence that the lagged adaptation phenomenon in men’s housework participation is not as evident in the period between 2002 and 2012 for the overall pattern of the association between gender attitudes and housework share. These findings are further discussed in the section analysing the regional results.

There is a significant period effect for women but not for men (see Model 3 in Tables 3, 4). The direction of association is in the predicted direction: within the period from 2002 to 2012, women lessened and men increased their housework participation, with other factors are held equal. The results in Model 4 in Tables 3, 4 reveal that most of the period effect among women can be attributed to the changing association between EGA and housework share. The results also show that the period effect among men, net of the interaction with the EGA, is negative (b = −4.041, SE = 1.725). This finding indicates that the overall mean of the housework performed by men have decreased in 2012 compared to 2002 when all else is held equal. The interaction terms between EGA and housework share show that the change in housework share was mostly driven by women and men with more egalitarian attitudes. This trend is characteristic of the transitional period.

To illustrate this finding, we summarized the marginal means for each level of egalitarian attitudes on housework participation among women (left panel) and men (right panel) in Figure 3. The figure uses a quadratic approximation for the association between egalitarianism and housework share based on Model 5 in Tables 3, 4 because quadratic approximations provide a more accurate prediction of the association between egalitarianism and housework share and it helps us reveal the pattern of lagged adaptation among men. The marginal means analysis reveals that the change in the association between EGA and housework share can be traced only for egalitarian women and men, whereas the change among more traditional women and men was not statistically significant. In addition, this might indicate that traditional women and men lag in adaptation compared to their more egalitarian counterparts (Gershuny et al., 2005), indicating a nascent polarization between egalitarian and traditional women and men and the transitional phase in the overall pattern among the analysed societies (Lesthaeghe, 2010). Moreover, the changes are significant for women who score average levels of egalitarianism and higher, whereas the results are marginally significant only for the most egalitarian men. These results confirm the lagged adaptation among men. We summarized the results for other control variables in Supplementary Appendix 3.

Overall, the results provide support for Hypothesis 1. A higher level of EGA motivates women to decrease their housework participation and men—to increase their share of housework participation. These results are indicative of the transitional period of the SDT where the changes among more egalitarian women and men precede the adaptation to the changing gender ideology among more traditional women and men (Lesthaeghe, 2010). The acute differences between more egalitarian and more traditional women and men revealed in Figure 4 provide evidence for this interpretation.

### Pre-transitional, Transitional, and Post-Transitional Welfare Regimes

The analysis of the association between EGA and housework share by regimes/regions confirm the post-transitional period among the socio-democratic regime (Scandinavian) countries. Thus, we find that Hypothesis 2 holds. The results summarized in Tables 5, 6 confirm that the association started to slow down in between 2002 and 2012.

**TABLE 5:** Summary of EGA random slopes by welfare regime and by years, women.

	Women, year 2002	N	Women, year 2012	N		t
Scandinavia	−2.036 (0.394)	783	−2.619 (0.471)	765	→	1.344
East Asia	−1.689 (0.478)	606	−2.936 (0.690)	474	↗	2.148^*^
Central Europe	−2.073 (0.397)	852	−2.797 (0.468)	1016	↗	2.073^*^
South Europe	−1.961 (0.464)	443	−3.137 (0.627)	503	↗	2.113^*^
Eastern Europe	−1.254 (0.430)	932	−1.522 (0.527)	1087	→	0.553
Liberal	−1.929 (0.414)	901	−3.315 (0.573)	829	↗	2.791^**^
Latin America	−1.547 (0.439)	879	−2.575 (0.589)	734	↗	2.005^*^

Standard errors in parentheses. ^*^ *p* < 0.05, ^**^ *p* < 0.01, ^***^ *p* < 0.001. All presented estimates are based on Models 1 and 2 in Tables 3, 4.

**TABLE 6:** Summary of EGA random slopes by welfare regime and by years, men.

	Men, year 2002	N	Men, year 2012	N		t
Scandinavia	2.698 (0.287)	700	1.801 (0.433)	683	↘	2.448^*^
East Asia	2.705 (0.307)	510	4.047 (0.841)	499	↗	−2.129^*^
Central Europe	2.802 (0.298)	517	1.575 (0.482)	771	↘	2.935^**^
South Europe	2.755 (0.306)	356	5.097 (0.671)	447	↗	−4.333^***^
Eastern Europe	2.636 (0.299)	734	0.851 (0.587)	929	↘	3.706^***^
Liberal	2.539 (0.298)	651	4.703 (0.624)	706	↗	−4.370^***^
Latin America	2.600 (0.301)	703	2.208 (0.991)	599	→	0.554

Standard errors in parentheses. ^*^ *p* < 0.05, ^**^ *p* < 0.01, ^***^ *p* < 0.001. All presented estimates are based on Models 1 and 2 in Tables 3, 4.

The consequences of the on-going SDT among women are apparent in other countries: East Asian, Conservative (Central European), Southern European, Liberal-regime, and Latin American countries. However, the results are not on a statistically significant level for Eastern Europe. As mentioned earlier, East European countries have mixed characteristics of the Conservative and the Social Democratic welfare regime types (Fenger, 2007). Therefore, we may find similarities between East European regimes and Scandinavian countries in the stage of SDT. This finding might suggest post-transitional period for some of the Eastern European countries, particularly the Baltic countries (Latvia). On the other hand, the findings may reflect a stalled progress in gender equality because of the reduction in social expenditure in the post-communist period, so that the SDT remained in pre-transitional period or an early stage of the transitional period in these countries. It might also indicate a reversal for more egalitarian women, which slowed down the transitional stage in this region. In the recent decade, Eastern European countries, particularly Russia, experienced a cultural return to more traditional gender roles, which also might have found its reflection in the present results.

The changes in the association are more complex for men. Within the ISSP data, the results for men are also consistent with Hypothesis 1 in East Asia, Southern Europe and Liberal states. As can be seen, the association between men’s gender attitudes and housework increases in these regimes/regions. On the other hand, we find that the difference between more traditional and egalitarian men are slowing down in Scandinavian and Central European regions, indicating that men are settling into the new equilibrium of the post-transitional stage, where more traditional men are catching up with more egalitarian ones. These results may suggest that men and women may have different pace in the adaption to more egalitarian housework share and gender attitudes in the SDT. We also find such deceleration in Eastern European and Latin American countries. However, the interpretation differs from the findings in Eastern Europe and Latin America, considering the political developments in the regions. We explain these findings in Eastern Europe and Latin America as the evidence of a potential reversal in these regions as a result of recent social shocks rather than of the SDT.

Overall, the results broadly support Hypotheses 2 and 3: the change in the association between gender attitudes and housework between 2002 and 2012 varied among the countries. Scandinavian countries appear to have already entered the post-transitional stage in the period.

The gender differences in the change in the association between gender attitudes and housework share within welfare regimes reveal that men and women may go through SDT at different paces and stages. For example, the magnitude of the association did not change for Scandinavian women but became weaker for Scandinavian men between 2002 and 2012. This indicates that Scandinavian women have already entered the post-transitional stage, while Scandinavian men are at later phase of the transitional stage. Similar patterns of change are observed in the case of Eastern European women and men. However, the gender differences in Eastern European countries are more likely due to political and social instabilities and the stagnation or the reversal of progresses in gender equality in the labour market and the domestic division of labour in some of these countries.

In Central European societies, the association between gender attitudes and housework share became stronger for women but weaker for men between 2002 and 2012. This indicates that women are at an earlier phase of the transitional stage than men. Men may not necessarily lag behind women in the adaptation in attitudes and housework. In addition, they may settle at a different level of gender egalitarianism in attitudes and gender roles than women.

In Latin American countries, the association between gender attitudes and housework share became stronger for women but remained unchanged for men between 2002 and 2012. This suggests that women in these countries have already entered the transitional stage but men remained in the pre-transitional stage.

## Conclusion

Our paper investigated whether gender ideology worked in an expected way in its effects on housework participation as the previous research suggests. Moreover, we connected the findings with the predictions of the three-stage transitional theory, introduced in the theoretical part of this paper. The results established that a more egalitarian outlook translated into less housework for women and more housework for men, and the association was stronger in the regions that were undergoing the transitional period. Using the ISSP data for 24 countries, we find that most analysed regions are in the transitional stage, where egalitarian attitudes are tied with more egalitarian housework division for both women and men. Women with more egalitarian views do significantly less housework, and more egalitarian men do more.

We also find for the overall pattern, net of the effects of the country context, also shows the evidence of the lagged adaptation for more traditional women and men, compared to their more gender-egalitarian counterparts (Gershuny et al., 2005). Thus, the gains in higher housework participation can be observed among women and men with higher levels of egalitarian views, whereas for more traditional men such a trend is not evident.

The analysis by welfare regimes showed that in countries of the Scandinavian region, the association between EGA and housework share has slowed down: the change in the slope of the association is not on a statistically significant level unlike in other regions. This result confirms both theories by Lesthaeghe (2010) and Esping-Andersen and Billari (2015). The same process is evident among men in Central European countries. The identified transitional states are as predicted by Esping-Andersen and Billari (2015) are in Southern Europe, Liberal regime countries, as well as East Asian countries. Therefore, even in countries of the East Asian region, where a more traditional division of housework is often reported, our findings discern the harbingers of the transitional stage. The frameworks are less likely to be able to explain the results for Latin American and Eastern European women and men, which experienced less stable political, economic, and social situations in the period between 2002 and 2012. These contradictory findings may suggest that the SDT is not always a linear process, but the changes it anticipates may be stagnated or even reversed.

Due to the limitations in the data used in this study, we have only examined changes in the association between housework share and gender attitudes between 2002 and 2012. Future research should examine the trend in association between housework and gender attitudes from 2002 to the early 2020s when data become available. The current data do not allow us to explore the association between housework and the spouse’s gender attitudes. We believe this is also a valuable avenue for future research.

## Data Availability

Publicly available datasets were analyzed in this study. The data that support the findings of this study are made available from the ISSP programme website: http://w.issp.org/data-download/by-year/.
