# Supplementary material for: Three-Stage Transitional Theory: Egalitarian Gender Attitudes and Housework Share in 24 Countries
Source: Front Sociol. 2021 Nov 5;6:700301. doi: 10.3389/fsoc.2021.700301 (PMC8607167; doi:10.3389/fsoc.2021.700301)
Supplement: Supplementary file 1 [file Table1.pdf]

Appendices

Appendix 1

Family is one of the main factors of the welfare regimes. It influences and is influenced by the welfare state and the labor market (Esping-Andersen 1999). Therefore, regime characteristics must be evident in how women and men divide their domestic work and in their gender attitudes because these are heavily influenced by the state and social gender ideology as well as the actual structure of the labor market.

This reflection of welfare regimes in gender attitudes and housework participation are not expected to be perfect because: (1) women's participation in housework maybe other than related to the economic or attitudinal choice and may not be an adequate reflection of women's relative insecurity in the labor market. (2) attitudes may have formed for historic and social reasons other than related to the welfare regimes.

We use hierarchical clustering on two main factors: gender attitudes and housework participation. For each country we create four variables for each factor by gender and by year. The resulting dendrogram is presented in Figure A1.

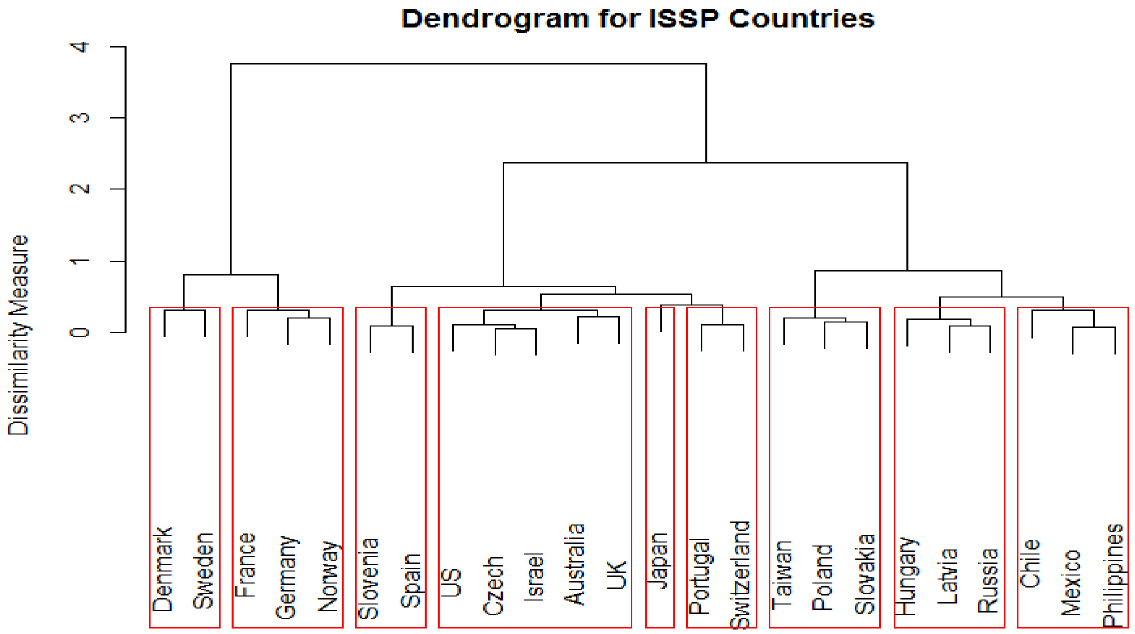

Figure A1 Hierarchical Cluster Analysis of ISSP Countries

Cluster analysis was more consistent in identifying Social-democratic, Conservative, Liberal regimes as well as a cluster for Latin American countries. It was less precise for countries of Southern Europe, Eastern Europe and East Asia.

The cluster analysis shows that if countries are clustered based on the mean values of gender attitudes and housework participation, Social-democratic and Conservative regime countries show more similarities. Anglo-Saxon countries form a distinct cluster with Israel and Czech Republic showing similarities with them. Two Latin American countries form a cluster, and the Philippines, the only Southeast-Asian country, are grouped with them.

Eastern European countries are more diverse. Slovenia shows more similarities with Spain, whereas Portugal forms a cluster with Switzerland – the most traditional Central-Western European

country. Post-soviet countries, Russia and Latvia, are grouped together and Hungary, the only non-Slavic language Eastern European country joins the group. Among East Asian countries, Japan stands alone, whereas Taiwan shows more similarities with Eastern European countries like Poland and Slovakia.

Appendix 2

Table. Summary of the 7-item Construct of Gender Ideology, Item Correlations

Items	*1*	*2*	*3*	*4*	*5*	*6*	*7*	
(1) warm relationship with children as a not working mom	-							
(2) Working mom: Preschool child is likely to suffer	.345***	-						
(3) Working woman: Family life suffers when woman has full-time job	.349***	.610***	-					
(4) What women really want is home and kids	.185***	.378***	.395***	-				
(5) Being housewife is as fulfilling as working for pay	.034***	.209***	.197***	.342***	-			
(6) Both should contribute to household income	.213***	.104***	.127***	.043***	.132***			
(7) Men's job earn money, women's job look after home	.240***	.395***	.394***	.492***	.300***	.161***		
Mean +- SE (weighted)	3.734 (0.006)	2.945 (0.006)	3.046 (0.006)	2.866 (0.006)	2.806 (0.006)	4.010 (0.005)	3.264 (0.006)	
Cronbach's alpha								.731

Adjusted coefficients; Robust standard errors in parentheses * $p < 0.05$, ** $p < 0.01$, *** $p < 0.001$

Appendix 3

Table A3. Random Intercept – Random Slopes Estimates for Housework Share Among Women, ISSP 2002-2012

	Model (1)	Model (2)	Model (3)	Model (4)	Model (5)

	2002	2012	Combined		
Egalitarian Gender Attitudes	-1.784***	-2.700***	-2.306***	-1.865***	3.712
	(0.427)	(0.497)	(0.424)	(0.483)	(2.357)
Year: 2012			-2.059***	0.909	6.094
			(0.352)	(1.533)	(5.339)
2012 # EGA				-0.872*	-4.196
				(0.438)	(3.212)
EGA # EGA					-0.839*
					(0.348)
2012 # EGA # EGA					0.503
					(0.469)
Employed	-3.890***	-2.319**	-2.938***	-2.930***	-2.967***
	(0.777)	(0.711)	(0.518)	(0.518)	(0.518)
Higher Income	1.089***	0.865***	0.933***	0.931***	0.939***
	(0.176)	(0.179)	(0.124)	(0.124)	(0.124)
Married	1.398^{+}	-0.414	0.211	0.227	0.203
	(0.782)	(0.725)	(0.527)	(0.527)	(0.526)
Education	-0.539***	-0.299***	-0.410***	-0.411***	-0.410***
	(0.084)	(0.078)	(0.057)	(0.057)	(0.057)
Lower PI Quartile	Ref.	Ref.	Ref.	Ref.	Ref.
25-50th PI Percentile	-0.981	-2.224**	-1.800**	-1.843**	-1.826**
	(0.839)	(0.784)	(0.566)	(0.567)	(0.566)
50-75th PI Percentile	-2.065*	-5.558***	-4.029***	-4.026***	-3.986***
	(0.896)	(0.857)	(0.614)	(0.614)	(0.614)
Upper PI Quartile	-4.689***	-7.736***	-6.450***	-6.457***	-6.378***
	(1.050)	(1.041)	(0.735)	(0.734)	(0.735)
Lower HI Quartile	Ref.	Ref.	Ref.	Ref.	Ref.
25-50th HI Percentile	-0.237	0.263	-0.030	-0.023	-0.027
	(0.865)	(0.911)	(0.626)	(0.626)	(0.626)
50-75th HI Percentile	0.205	1.732^{+}	0.844	0.844	0.847
	(0.895)	(0.950)	(0.650)	(0.649)	(0.649)
Upper HI Quartile	-0.246	2.155*	0.891	0.905	0.900
	(0.996)	(1.053)	(0.722)	(0.722)	(0.722)
Outsourcing	-1.377^{+}	-0.603	-1.087^{+}	-1.065^{+}	-1.058^{+}
	(0.781)	(0.817)	(0.564)	(0.564)	(0.564)
20-24 in 2002	Ref.	Ref.	Ref.	Ref.	Ref.
25-29 in 2002	1.949^{+}	0.747	1.085	1.065	1.071
	(1.167)	(0.991)	(0.749)	(0.749)	(0.749)
30-34 in 2002	2.419*	1.838^{+}	1.908**	1.881*	1.889*
	(1.147)	(0.990)	(0.739)	(0.739)	(0.739)
35-39 in 2002	3.133**	1.588	2.128**	2.122**	2.110**
	(1.168)	(0.994)	(0.747)	(0.747)	(0.747)
40-44 in 2002	4.574***	4.335***	4.266***	4.260***	4.256***
	(1.165)	(1.010)	(0.750)	(0.750)	(0.750)
45-49 in 2002	4.344***	3.077**	3.578***	3.579***	3.567***
	(1.179)	(1.055)	(0.775)	(0.775)	(0.775)
50-54 in 2002	5.186***	2.484*	3.658***	3.666***	3.663***
	(1.193)	(1.079)	(0.790)	(0.790)	(0.790)
Children under 17	0.676*	0.129	0.418^{+}	0.434^{+}	0.434^{+}
	(0.344)	(0.359)	(0.244)	(0.244)	(0.244)
Spouse: Employed	4.641***	4.157***	4.453***	4.466***	4.473***
	(0.768)	(0.737)	(0.525)	(0.525)	(0.525)
Household Size	0.517^{+}	0.544^{+}	0.540**	0.538**	0.535**
	(0.269)	(0.288)	(0.196)	(0.196)	(0.196)
Country	Yes	Yes	Yes	Yes	Yes
Constant	75.826***	70.918***	73.452***	72.050***	63.047***
	(3.042)	(2.796)	(2.136)	(2.258)	(4.272)
Observations	5396	5408	10804	10804	10804

Log-likelihood	-23003.686	-23173.736	-46214.546	-46212.657	-46209.181
Chi Square	1215.594	865.360	1909.243	1902.340	1999.194
d.f.	44.000	44.000	45.000	46.000	48.000

Standard errors in parentheses. $^{+}$ $p < 0.10$, * $p < 0.05$, ** $p < 0.01$, *** $p < 0.001$

Table A4. Random Intercept - Random Slopes Estimates for Housework Share Among Men, ISSP 2002-2012

	Model (1)	Model (2)	Model (3)	Model (4)	Model (5)
Egalitarian Gender Attitudes	2.677***	2.897***	2.907***	2.223***	4.959^{+}
	(0.451)	(0.776)	(0.457)	(0.530)	(2.696)
Year: 2012			0.172	-4.041*	10.641^{+}
			(0.397)	(1.725)	(5.859)
2012 # EGA				1.282*	-7.995*
				(0.511)	(3.596)
EGA # EGA					-0.416
					(0.408)
2012 # EGA # EGA					1.392**
					(0.537)
Employed	-4.696***	-6.172***	-5.667***	-5.713***	-5.712***
	(1.280)	(0.980)	(0.773)	(0.773)	(0.773)
Higher Income	2.226***	2.308***	2.278***	2.279***	2.278***
	(0.250)	(0.231)	(0.170)	(0.170)	(0.170)
Married	-5.030***	-4.132***	-4.214***	-4.231***	-4.233***
	(0.919)	(0.804)	(0.601)	(0.601)	(0.601)
Education	0.291**	0.523***	0.413***	0.415***	0.418***
	(0.090)	(0.080)	(0.060)	(0.060)	(0.060)
Lower PI Quartile	Ref.	Ref.	Ref.	Ref.	Ref.
25-50th PI Percentile	-0.432	2.826*	1.466	1.516	1.565
	(1.630)	(1.285)	(0.999)	(0.999)	(0.998)
50-75th PI Percentile	-1.828	0.170	-0.307	-0.291	-0.288
	(1.640)	(1.328)	(1.022)	(1.022)	(1.022)
Upper PI Quartile	-3.999*	-2.059	-2.521*	-2.507*	-2.522*
	(1.751)	(1.454)	(1.108)	(1.107)	(1.107)
Lower HI Quartile	Ref.	Ref.	Ref.	Ref.	Ref.
25-50th HI Percentile	-0.166	0.509	-0.152	-0.169	-0.102
	(1.152)	(1.115)	(0.801)	(0.800)	(0.800)
50-75th HI Percentile	1.146	0.873	0.549	0.561	0.648
	(1.245)	(1.211)	(0.865)	(0.865)	(0.865)
Upper HI Quartile	2.807*	1.714	1.765^{+}	1.763^{+}	1.837^{+}
	(1.392)	(1.338)	(0.961)	(0.960)	(0.960)
Outsourcing	-1.278	0.824	-0.051	-0.058	-0.071
	(0.966)	(0.910)	(0.665)	(0.664)	(0.664)
20-24 in 2002	Ref.	Ref.	Ref.	Ref.	Ref.
25-29 in 2002	-1.146	2.202^{+}	1.082	1.113	1.112
	(1.695)	(1.196)	(0.975)	(0.974)	(0.974)
30-34 in 2002	-2.366	0.335	-0.658	-0.592	-0.593
	(1.637)	(1.196)	(0.953)	(0.953)	(0.952)
35-39 in 2002	-2.196	-0.386	-0.939	-0.882	-0.848
	(1.642)	(1.189)	(0.951)	(0.951)	(0.950)
40-44 in 2002	-3.300*	-1.306	-1.939*	-1.921*	-1.926*
	(1.632)	(1.175)	(0.941)	(0.941)	(0.941)
45-49 in 2002	-3.745*	-1.365	-2.230*	-2.189*	-2.181*
	(1.634)	(1.188)	(0.949)	(0.948)	(0.948)
50-54 in 2002	-4.659**	-1.718	-2.792**	-2.805**	-2.803**
	(1.640)	(1.207)	(0.960)	(0.960)	(0.959)
Children under 17	-0.898*	-0.490	-0.651*	-0.675**	-0.677**

	(0.388)	(0.358)	(0.259)	(0.259)	(0.259)
Spouse: Employed	5.105***	5.434***	5.237***	5.253***	5.263***
	(0.707)	(0.668)	(0.479)	(0.479)	(0.479)
Household Size	-0.418	-0.229	-0.351+	-0.342+	-0.349+
	(0.306)	(0.279)	(0.205)	(0.205)	(0.205)
Country	YES	YES	YES	YES	YES
Constant	31.279***	19.102***	24.295***	26.436***	22.107***
	(3.702)	(3.251)	(2.378)	(2.523)	(4.787)
Observations	4171	4634	8805	8805	8805
Log-likelihood	-17848.415	-19834.218	-37730.291	-37727.149	-37723.173
Chi Square	1262.698	929.516	2090.582	2099.129	2124.940
d.f.	44.000	44.000	45.000	46.000	48.000

Standard errors in parentheses. + $p < 0.10$, * $p < 0.05$, ** $p < 0.01$, *** $p < 0.001$
